# How to Clean the Hands

**Published:** 1888-04

**Authors:** 


### How to Clean the Hands.

Dr. P. Furbringer, of Berlin, has published a pamphlet upon the disinfection of the surgeon's hands and finger-nails. From an abstract of this in the *Med. Press and Circular*, Jan. 11, 1888, we take the following, which the author recommends as the speediest method of cleansing the hands, consistent with security.

1. The nails are to be cleansed dry (scraped or rubbed) from all visible dirt.

2. The hands are to be thoroughly scrubbed with a brush for a minute and a half in hot water and soap, special attention being paid to the edges of the nails.

3. They are then to be washed for one minute in alcohol (not under 80 per cent.), and then, before drying.

4. They are to be put into the disinfecting solution, 2 per cent. sublimate or 2 per cent. carbolic solution, and here to be thoroughly cleansed for another minute.

He claims for this method (1) Certainty of disinfection. (2) Saving of time. (3) Saving of the hands. (4) Smaller expense in the use of the sublimate.

---

Aceto-tartrate of Aluminum, the New Anti-septic.—This substance that Athenstaldt recommends as an anti-septic, is prepared by mixing a $\frac{5}{100}$ solution of acetate of ammonium and a $\frac{2}{100}$ solution of tartaric acid, the double salt formed being obtained by evaporation. Aceto-tartrate of aluminum is a brilliant, colorless substance resembling gum arabic. It dissolves readily in water, has the odor of acetic acid, and has an acid, slightly astringent, but not disagreeable taste. It is soluable in equal parts of water, and the solution remains clear when heated, but is precipitated by alcohol. It becomes less soluble when exposed to the air on account of the loss of the acetic acid radicle of the double salt. Athenstaldt recommends it as a harmless antiseptic.—*Journal de Medicine*, Dec. 18, 1887.

